# Purine-Metabolizing Ectoenzymes Control IL-8 Production in Human Colon HT-29 Cells

**DOI:** 10.1155/2014/879895

**Published:** 2014-07-23

**Authors:** Fariborz Bahrami, Filip Kukulski, Joanna Lecka, Alain Tremblay, Julie Pelletier, Liliana Rockenbach, Jean Sévigny

**Affiliations:** ^1^Département de Microbiologie-Infectiologie et d'Immunologie, Faculté de Médecine, Université Laval, Québec, QC, Canada G1V 0A6; ^2^Centre de Recherche du CHU de Québec, 2705 Boulevard Laurier, Local T1-49, Québec, QC, Canada G1V 4G2

## Abstract

Interleukin-8 (IL-8) plays key roles in both chronic inflammatory diseases and tumor modulation. We previously observed that IL-8 secretion and function can be modulated by nucleotide (P2) receptors. Here we investigated whether IL-8 release by intestinal epithelial HT-29 cells, a cancer cell line, is modulated by extracellular nucleotide metabolism. We first identified that HT-29 cells regulated adenosine and adenine nucleotide concentration at their surface by the expression of the ectoenzymes NTPDase2, ecto-5′-nucleotidase, and adenylate kinase. The expression of the ectoenzymes was evaluated by RT-PCR, qPCR, and immunoblotting, and their activity was analyzed by RP-HPLC of the products and by detection of P_i_ produced from the hydrolysis of ATP, ADP, and AMP. In response to poly (I:C), with or without ATP and/or ADP, HT-29 cells released IL-8 and this secretion was modulated by the presence of NTPDase2 and adenylate kinase. Taken together, these results demonstrate the presence of 3 ectoenzymes at the surface of HT-29 cells that control nucleotide levels and adenosine production (NTPDase2, ecto-5′-nucleotidase and adenylate kinase) and that P2 receptor-mediated signaling controls IL-8 release in HT-29 cells which is modulated by the presence of NTPDase2 and adenylate kinase.

## 1. Introduction

Inflammation is a major contributor to the development and progression of many human cancers [1] and is obviously a key constituent of inflammatory diseases such as inflammatory bowel diseases (IBD) [2–4]. Indeed, a number of chronic inflammatory conditions increase the risk of developing cancers [5]. For instance, IBD is associated with an increased risk of colon cancer development [6, 7]. In addition, the long-term use of anti-inflammatory drugs such as aspirin decreases the risk of several cancer types [8].

Interleukin-8 (IL-8) or CXCL8 is a proinflammatory chemokine originally identified as a neutrophil chemoattractant [9], which is an important contributor to the induction of innate immunity [10]. Accordingly, IL-8 has been implicated in a number of inflammatory diseases such as IBD [11, 12]. Elevated IL-8 signaling has also been observed within the tumor microenvironment of numerous cancers where it enhances tumor progression via the activation of pathways that promote proliferation, angiogenesis, migration, invasion, and cell survival [13, 14]. Altogether, this suggests that inhibition of IL-8 production could be a potential treatment for both chronic inflammatory diseases and cancer [13, 15]. Therefore, a better understanding of the mechanisms that drive or mediate IL-8 release is imperative.

We have previously observed that IL-8 secretion, and even function, can be controlled by nucleotide receptors [16–18]. Extracellular nucleotides (e.g., ATP, ADP, UTP, and UDP) are secreted by host cells in response to injury, such as in conditions of inflammation, and act as danger signals (alarmins) and damage-associated molecular patterns (DAMPs). These substances initiate the host immune responses [19–21] by activating specific P2 receptors [22]. The concentration of P2 receptor agonists is regulated by ectoenzymes that metabolize nucleotides [23–26]. While ectonucleotidases such as nucleoside triphosphate diphosphohydrolases (NTPDases) generally terminate P2 receptor activation [24], nucleotide kinases such as adenylate kinase (ADK) may potentiate P2 activation by regenerating the ligand of these receptors from the products of ectonucleotidases [27–29].

In this work, we used HT-29 colon cancer cell line as a model of intestinal epithelial cells (used in IBD models) as well as a model of cancer cells to investigate if, in such cells, ectoenzymes that modulate nucleotide metabolism can control IL-8 secretion. Indeed, HT-29 cells express and secrete IL-8 in response to diverse stimuli [30, 31] such as TLR3 agonists [32]. They also express functional receptors that respond to ATP and/or UTP [33–35] as well as to adenosine [36–42] that are involved in several functions including cell growth and differentiation, and IL-8 release. Our initial objective was therefore to characterize the expression of nucleotide metabolizing ectoenzymes. We identified 3 of these enzymes and 2 of them affected IL-8 release in our system: NTPDase2 which is a predominant ATPase [43] and ADK that catalyzes the reversible transphosphorylation reaction leading to ATP and AMP production from two molecules of ADP as substrate [23]. The ecto-5′-nucleotidase that hydrolyses AMP into adenosine [44, 45] was also highly expressed in these cells.

## 2. Materials and Methods

### 2.1. Materials

DMEM/F-12 growth medium, Glutamax, Hu IL-8 Cytoset ELISA kit, PureLink Genomic DNA mini kit, Quant-iT RNA BR assay kit, NuPAGE Novex 4–12% Bis-Tris gel, TRIzol reagent, DNAse1-RNAse-free (AM2222), Superscript III reverse transcriptase, RNAseOUT recombinant Ribonuclease inhibitor, dNTP, DTT, aprotinin, Lipofectamine, microAMp optical 384 well reaction plate, custom-made primers, and 1 kb plus DNA ladder were purchased from Life Technologies (Burlington, ON, Canada). Normocin was obtained from InvivoGen (San Diego, CA, USA). ATP, ADP, AMP, adenosine, ATP-*γ*-S, suramin, diadenosine pentaphosphate (Ap_5_A), malachite green, and random nonamers (R7647) were purchased from Sigma. Millex GP syringe-driven filter unit 0.22 *µ*m and Immobilon-P membrane were from Millipore (Billerine, MA, USA). Hank's balanced salt solution (HBSS) with Ca^2+^ and Mg^2+^, Hepes, and antibiotic-antimycotic solutions was from Wisent (St. Bruno, QC, Canada). RNeasy mini kit and QuantiTect Reverse Transcription kit were from Qiagen (Mississauga, ON, Canada). Taq polymerase was obtained from New England Biolabs (Ipswich, MA, USA) and FastStart Universal SYBR Green Master (Rox) was from Roche (Mannheim, Germany).

### 2.2. Cell Culture and Treatment

HT-29 (ATCC^®^ HTB-38^TM^) human colon adenocarcinoma cell line was purchased from the American Type Culture Collection and maintained in monolayer cultures in DMEM/F-12 growth medium supplemented with Glutamax (2 mM), antibiotic-antimycotic solution (1X), Hepes (25 mM), Normocin (used as an antimycoplasma reagent, 100 *μ*g/mL), and 10% heat-inactivated fetal bovine serum at 37°C in a 95% air: 5% CO_2_ atmosphere. Cells were regularly monitored for the presence of* Mycoplasma *spp. by means of a conventional PCR test [47] using 5 *μ*g of extracted genomic DNA (PureLink genomic DNA mini kit) as a template. The cells from passages 2-3 were seeded (2 × 10^6^/well) in 24-well plates containing 1 mL medium. For cell counting and subculturing, cells were dispersed with a 0.25% trypsin solution. Cell viability always exceeded 95%, as measured by Trypan blue dye exclusion.

### 2.3. IL-8 Production Assays

HT-29 cells were stimulated with suboptimal concentration of poly (I:C) alone or in presence of nucleotides with or without Ap_5_A as an ADK inhibitor. Stimulations were carried out for 18 h. Media were then collected and centrifuged to remove detached cells and were analyzed for the detection of human IL-8 using the Human IL-8 CytoSet ELISA kit.

### 2.4. RP-HPLC

HT-29 cells were incubated with ADP or ATP (both at 100 *µ*M) in Hank's balanced salt solution (HBSS) containing Ca^2+^ and Mg^2+^. Where indicated, these incubations were carried out in the presence of the ADK inhibitor, Ap_5_A (10 *µ*M). At the indicated time points, 100 *µ*L aliquots of medium were sampled, deproteinized with an equal volume of PCA (1 M), and neutralized with KOH (1 M), with all solutions being kept at 4°C. Analysis of the reaction products was performed by RP-HPLC using 15 cm × 3.6 mm, 3 *µ*m SUPELCOSIL LC-18-T columns (Supelco) as described previously [43].

### 2.5. RT-PCR

Total RNA was purified from HT-29 cells using the RNeasy mini kit and quantified with a Quant-iT RNA BR assay kit and Qubit fluorometer (Life Technologies). The cDNA was prepared using QuantiTect Reverse Transcription kit. For NTPDase screening, semiquantitative amplifications were done with 1 *µ*L cDNA prepared from 3 *µ*g total RNA and Taq polymerase using a PTC-200 Peltier Thermal Cycler in 25 *μ*L reaction volumes and the following program: (i) 2 min at 94°C and (ii) 20 cycles of 1 min at 94°C, 1 min at 75°C (and then decreasing by 1°C/cycle), and 1 min at 72°C, followed by (iii) 30 cycles of 1 min at 94°C, 1 min at 60°C, and 1 min at 72°C and (iv) a final 7 min step at 72°C. For the positive controls, 10 pg of miniprep DNA from the expression vectors used for cloning NTPDase1, -3, and -8 were used as templates [48] and due to the location of the primers, 50 ng of HT-29 genomic DNA was used for NTPDase2. For the negative controls, water was used as template. For P2Y receptor screening, using similar conditions for preparation, the program was (i) 2 min at 94°C and (ii) 20 cycles of 1 min at 94°C, 1 min at 72°C (and then decreasing by 1°C/cycle), and 1 min at 72°C, followed by (iii) 20 cycles of 1 min at 94°C, 1 min at 57°C, and 1 min at 72°C and (iv) a final 7 min step at 72°C. For human AK1*β* amplification, total RNA was extracted and quantified as above, and the cDNA was prepared using 1 *μ*g RNA and Superscript III reverse transcriptase, as specified by the manufacturer. Semiquantitative amplifications were performed as above, except that the amplification program used was (i) 2 min at 94°C, (ii) 35 cycles of 30 sec at 94°C, 30 sec at 66.1°C, and 30 sec at 72°C, and (iii) a final 7 min at 72°C. Sequencing of the amplicons was performed by automated DNA sequencing at the* Plateforme de Génomique, Protéomique et Bio-informatique, CRCHU, Université Laval*.

### 2.6. qPCR

Total RNA was purified from HT-29 cells using TRIzol reagent and following the manufacturer's instructions. RNA was quantified with a Quant-iT RNA BR assay kit and Qubit fluorometer. For synthesis of cDNA, 2 *µ*g of RNA was treated for 15 min at 20°C with 2 units of DNase I (RNase-free) in a volume of 10 *μ*L, to remove contaminating DNA, followed by heat inactivation of the enzyme at 65°C for 10 min with 1 *µ*L of 25 mM EDTA. Treated RNA was annealed with 1 *µ*L random nonamers with 1 *µ*L dNTP (10 mM) and 1 *µ*L of water and heated at 65°C for 5 min and 1 min at 2°C in a PTC-200 Peltier thermal cycler. The cDNA was done with 1 *µ*L of Superscript III reverse transcriptase, 1 *µ*L of RNAseOUT, 1 *µ*L of 0.1 M DTT, and 4 *µ*L of 5X First-Strand Buffer, incubated in a PTC-200 Peltier Thermal cycler at 50°C for 60 min, and inactivated at 70°C for 15 min. For NTPDase mRNA evaluation, quantitative amplifications were done with 1 *µ*L cDNA, 5 *µ*L FastStart Universal SYBR Green Master (ROX), and 1 *µ*L of specific primers (3 *µ*M) in a MicroAMp optical 384-well reaction plate and using an Applied Biosystems 7900HT Fast Real-Time PCR system in 10 *μ*L reaction volumes and the following program: (i) 2 min at 50°C, (ii) 10 min at 95°C, (iii) 40 cycles of 15 sec at 95°C, 1 min at 60°C, and (iv) a dissociation stage. For the standard curve, 10 copies to 10^8^ copies of amplified fragment were used. For the negative controls, water was used as template. Each quantification was normalized with GAPDH. The primers used in this study for both RT-PCR and qRT-PCR are presented in Table 1.

### 2.7. Cell Transfection and Protein Preparation

HEK 293 cells were cultured and transfected in 10 cm plate using Lipofectamine as previously described [43]. Briefly, 80%–90% confluent cells were incubated for 5 h at 37°C in Dulbecco's modified Eagle's medium (DMEM) in the absence of fetal bovine serum (FBS) with 6 *µ*g of plasmid DNA encoding for human ecto-5′-nucleotidase (GenBank accession no. NM_002526) previously described [49] and 24 *µ*L of Lipofectamine reagent. The reaction was stopped by the addition of an equal volume of DMEM containing 20% FBS and the cells were harvested 48 h later. For the preparation of protein extracts, transfected cells or HT-29 cells were washed three times with Tris-saline buffer at 4°C collected by scraping in the harvesting buffer (95 mM NaCl, 0.1 mM PMSF, and 45 mM Tris at pH 7.5) and washed twice by 300 ×g centrifugation for 10 min at 4°C. Cells were resuspended in the harvesting buffer containing 10 *µ*g/mL aprotinin and sonicated. Nucleus and cellular debris were discarded by centrifugation at 600 ×g for 5 min at 4°C and the supernatant (crude protein extract) was aliquoted and stored at −80°C until used for experiments. Proteins concentrations were determined with a Quant-iT protein assay kit and Qubit fluorometer (Life Technologies).

### 2.8. Immunoblotting and Antibodies

Protein extract was resolved on a NuPAGE Novex 4–12% Bis-Tris gel and transferred to an Immobilon-P membrane by electroblotting according to the manufacturer's instructions (Millipore). The membrane was probed with the following antibodies: mouse anti-human NTPDase1 (BU61, Ancell Corporation (Bayport, MN, USA)), guinea pig anti-human NTPDase1 (hN1-1_c_I_5_), mouse monoclonal anti-human NTPDase2 (hN2-B2_s_ and hN2-H9_s_), guinea pig anti-human NTPDase3 (hN3-1_c_I_5_), mouse monoclonal anti-human NTPDase3 (hN3-B3_s_), mouse anti-human NTPDase8 (hN8-6_s_I_6_), mouse monoclonal anti-human NTPDase8 (hN8-D7_s_), rabbit anti-human ecto-5′-nucleotidase (h5′NT-2_L_I_5_), guinea pig anti-human ecto-5′-nucleotidase (h5′NT-2_c_I_5_), and the mouse monoclonal anti-human ecto-5′-nucleotidase (clone 4G4, Hycult Biotech, Plymouth Meeting, PA, USA). All antibodies, except the mouse monoclonal anti-human NTPDase1 BU61 and the mouse anti-human ecto-5′-nucleotidase clone 4G4, were produced by cDNA immunization in our laboratory and their specificities were confirmed by immunoblots and immunohistochemistry [48, 50, 51]. These antibodies are available at http://ectonucleotidases-ab.com/. The secondary horseradish peroxidase-conjugated antibodies used were goat anti-mouse (Jackson Immuno Research Laboratories Inc., West Grove, PA, USA), donkey anti-rabbit (GE Healthcare Life Sciences, Baie d'Urfe, Québec, Canada), and goat anti-guinea pig (Santa Cruz Biotechnology, Dallas, TX, USA). The blots were developed with the Western Lightning Plus-ECL reagent (PerkinElmer Life and Analytical Sciences, Waltham, MA, USA).

### 2.9. Enzymatic Reactions

Enzyme activity was evaluated as described [43] in 0.2 mL of Tris-Ringer buffer (in mM: 120 NaCl, 5 KCl, 2.5 CaCl_2_, 1.2 MgSO_4_, 25 NaHCO_3_, 5 D-glucose, and 80 Tris; pH 7.4) at 37°C. HT-29 lysates were added to the incubation mixture and preincubated at 37°C for 3 min. The reaction was initiated by the addition of 500 *µ*M ATP, ADP, or AMP and stopped after 10–15 min with 50 *µ*L of malachite green reagent. The activity at the cell surface was measured with confluent HT-29 cells in 24-well plates (about 200,000 cells per well). Cells were maintained in Dulbecco's modified Eagle medium nutrient mixture F-12 (DMEM-F12) supplemented with 10% FBS until conducting the activity assay that was performed in the buffer indicated above. The reactions were initiated as above and stopped by transferring an aliquot of the reaction mixture to a tube containing the malachite green reagent. Net cell-bound enzyme activity was calculated after subtracting the value measured in the control cell reaction mixture where the substrate was added after the malachite green reagent. Released inorganic phosphate (P_i_) was measured at 630 nm according to Baykov et al. [52]. All experiments were performed in triplicate.

One unit of enzymatic activity corresponds to the release of 1 *μ*mol P_i_/(min·mg of protein) or 1 *μ*mol P_i_/min/well at 37°C for protein extracts and intact cells, respectively.

### 2.10. Statistical Analysis

Two-tailed Student's *t*-test was performed using the Microsoft 2007 Excel software. *P* < 0.05 was considered statistically significant.

## 3. Results

### 3.1. HT-29 Cells Express Purine-Metabolizing Ectoenzymes

**Figure 1:**
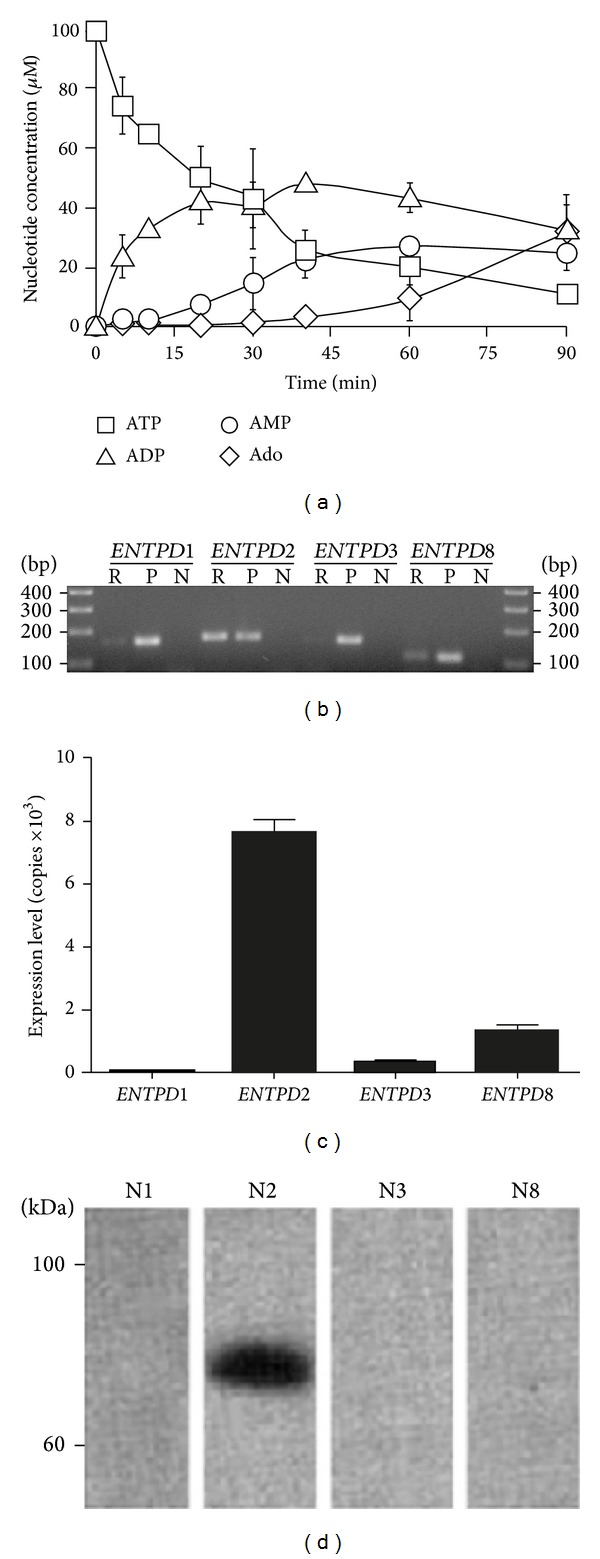
NTPDase2 is expressed by HT-29 cells. (a) HT-29 cells were incubated with ATP for 90 min and their supernatants were then subjected to RP-HPLC to quantify the various ATP degradation products (*n* = 3). (b) The expression of various human NTPDase genes was analyzed by semiquantitative RT-PCR where R, P, and N represent the reaction with HT-29 cells, the positive controls, and the negative controls, respectively, and (c) by quantitative RT-PCR (*n* = 5). (d) The expression of NTPDase proteins in HT-29 cells was analyzed by immunoblotting using specific antibodies to human NTPDase1, -2, -3, and -8, N1, N2, N3, and N8, respectively. A representative experiment of *n* = 4 is shown.

Our initial goal was to define the ectonucleotidases expressed in HT-29 cells. This was first done by investigating the metabolism of extracellular ATP and ADP by these cells. The analysis of ATP hydrolysis products showed a significant accumulation of ADP (Figure 1(a)), which fits the profile expected for NTPDase2 activity [43]. In agreement, semiquantitative PCR using primers specific to human NTPDase members expressed at the plasma membrane, namely, NTPDase1, -2, -3, and -8, showed high expression of NTPDase2 in HT-29 cells (Figure 1(b)). This was further confirmed by quantitative RT-PCR (Figure 1(c)). Immunoblotting experiments using 2 different sets of antibodies to human NTPDase1, -2, -3, and -8 with protein samples extracted from HT-29 cells were consistent with the PCR results and confirmed the predominant presence of NTPDase2 in these cells (Figure 1(d) for one set of antibody and data not shown for the guinea pig anti-human NTPDase1 (hN1-1_c_I_5_), mouse monoclonal NTPDase2 (hN2-H9_s_), mouse monoclonal NTPDase3 (hN3-B3_s_), and mouse monoclonal NTPDase8 (hN8-D7_s_) antibodies; see Section 2).

**Figure 2:**
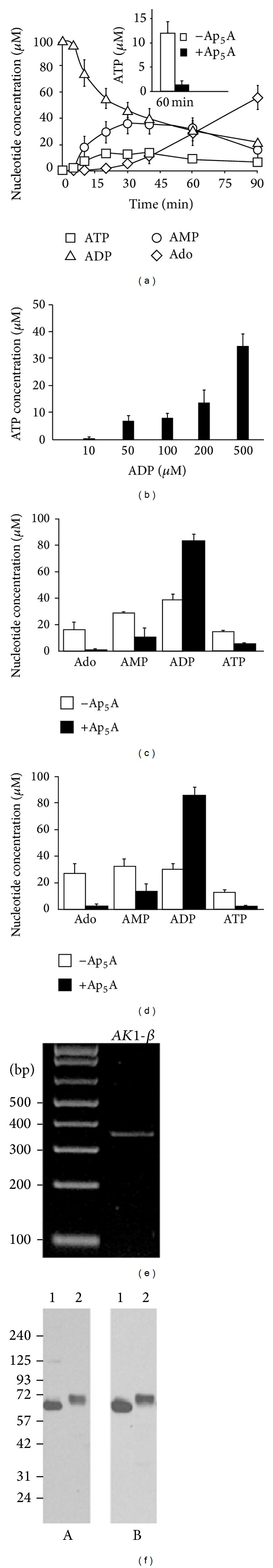
Adenylate kinase and ecto-5′-nucleotidase are expressed by HT-29 cells. (a) HT-29 cells were incubated with ADP for 90 min and their supernatants were subjected to RP-HPLC for the determination of purines and ADK activity (*n* = 3). Inset shows the effect of the ADK inhibitor, Ap_5_A, on ATP production by HT-29 cells after 1 h (*n* = 3). (b) Supernatants from HT-29 cells incubated for 30 min with various concentrations of ADP were subjected to RP-HPLC to determine ATP production (*n* = 3). ((c), (d)) Supernatants from HT-29 cells incubated for 30 min with either 100 *µ*M ATP ((c) *n* = 2) or ADP ((d) *n* = 3), in the presence of 10 *µ*M Ap_5_A or control vehicle, were subjected to RP-HPLC for the determination of adenine nucleotide and adenosine byproducts. (e) Semiquantitative RT-PCR analysis on HT-29 cell line showing the presence of the membrane associated adenylate kinase. A representative experiment of *n* = 2 is presented. (f) The expression of ecto-5′-nucleotidase protein was detected by immunoblotting with the rabbit anti-human ecto-5′-nucleotidase h5′NT-2_L_I_5_ ((A) on the left side) and with the commercial antibody clone 4G4 ((B) on right side). For panels (A) and (B), lane 1 represents the positive control of protein extract (6 *µ*g) from HEK 293 cells transiently transfected with a human ecto-5′-Nucleotidase expression vector and lane 2 protein extract (20 *µ*g) from HT-29.

RP-HPLC analyses further revealed that HT-29 cells have the ability to produce ATP when incubated with ADP (Figure 2(a)). This implied the presence of an adenylate kinase (ADK) activity, which catalyzes the reversible reaction: 2ADP ⇌ ATP + AMP. The amount of ATP produced varied according to ADP concentration (Figure 2(b)). To confirm that this activity belonged to ecto-ADK, we tested whether Ap_5_A, a specific inhibitor of this enzyme, might affect ATP production. As shown in the inset of Figure 2(a), the production of ATP from ADP was inhibited by Ap_5_A. Semiquantitative RT-PCR using a published set of primers [46] (data not shown) as well as a home-made set designed from EST clone 781374 (NCBI accession number: AA430294; cf. Table 1), together with the subsequent sequencing of the amplicons, showed that HT-29 cells express* AK1* *β* , which encodes the plasma membrane-localized isoform of ADK (Figure 2(e)).

Interestingly, the presence of ADK activity at the surface of HT-29 cells allowed adenosine production in the presence of either ATP (hydrolyzed to ADP by NTPDase2) or ADP. Indeed, adenosine production was prevented by the ADK inhibitor Ap_5_A (Figures 2(c) and 2(d)). As ATP and ADP, adenosine is of important biological value due to the various functions affected by this P1 receptor ligand. The production of adenosine cannot be explained by neither NTPDase2 nor ADK alone. Therefore the data presented in Figures 1(a), 2(a), 2(c), and 2(d) suggested the presence of ecto-5′-nucleotidase which was confirmed by 3 different antibodies that showed the same immunoreactive band (Figure 2(f); data not shown for the guinea pig anti-human ecto-5′-nucleotidase antibody; see Section 2). Finally, the hydrolysis of ATP, ADP, and AMP was also evaluated at the surface of HT-29 cells as well as with protein extracts which confirmed the presence of enzymes able to hydrolyze ATP and AMP as substrate (Table 2), in agreement with the presence of NTPDase2 and ecto-5′-nucleotidase.

### 3.2. NTPDase2 and ADK Affect IL-8 Production

We then addressed the hypothesis that the purine-metabolizing enzymes expressed at the surface of HT-29 cells can affect the release of IL-8. As ATP and ADP alone did not activate IL-8 release, these assays were performed in the presence of a suboptimal concentration of poly (I:C) which is a TLR3 agonist. TLR3 activation was selected for the following reasons. TLR3 activation stimulates IL-8 release in HT-29 cells [32] in a nucleotide dependent manner (manuscript in preparation). In addition, TLR3 was found to be involved in different cancers [53, 54] and was also reported to affect intestinal inflammation in a complex manner depending on the conditions, either protecting from inflammation or causing epithelial destruction [55–59].

**Figure 3:**
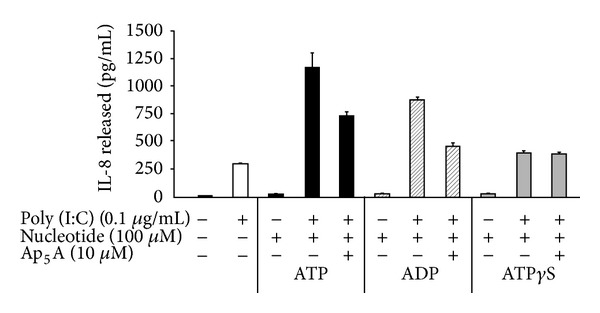
NTPDase2 and ADK modulate IL-8 production in HT-29 cells. HT-29 cells were stimulated for 18 h with suboptimal concentration of poly (I:C) in combination with ATP, ADP, or the nonhydrolyzable ATP analog, ATP-*γ*-S, in the presence or absence of the ADK inhibitor, Ap_5_A. The cell supernatants were then analyzed for IL-8 by ELISA (*n* = 3).

In agreement with a role of ADK in IL-8 release by ATP- or ADP-stimulated HT-29 cells, Ap_5_A significantly diminished IL-8 release induced by either nucleotide (Figure 3). As expected, Ap_5_A did not affect IL-8 release triggered by ATP-*γ*-S (Figure 3), a nonhydrolysable ATP analogue, suggesting that the reduction in ATP-induced IL-8 secretion seen in the presence of the ADK inhibitor was due to the hydrolysis of ATP to ADP catalyzed by NTPDase2. This experiment also confirmed that the effect of Ap_5_A on IL-8 release was specific to ADK inhibition as Ap_5_A did not affect the poor induction of IL-8 release by ATP-*γ*-S. In this system, adenosine did not affect IL-8 release when HT-29 cells were stimulated with the TLR3 agonist poly (I:C) (data not shown), suggesting that, in these conditions with this cell line, ecto-5′-nucleotidase did not affect IL-8 production and release.

### 3.3. HT-29 Cells Express P2Y Receptors

**Figure 4:**
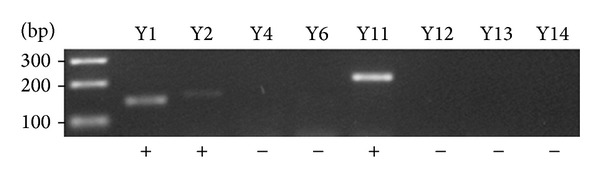
Comparative expression of human P2Y receptors in HT-29 cells by semiquantitative RT-PCR. A representative experiment of *n* = 2 is shown. Presence (+) of P2Y_1_, P2Y_2_, and P2Y_11_ was detected while the other P2Y receptors were not detected (−).

We then analyzed the repertoire of P2Y receptors expressed in HT-29 cells by semiquantitative PCR. As seen in Figure 4, HT-29 cells express P2Y_1_, P2Y_2_, and P2Y_11_. No, or very little, mRNA expression could be detected for the other P2Y receptors.

## 4. Discussion

The production of IL-8 by HT-29 cells was affected by two ectoenzymes present at the surface of these cells, namely, ADK and NTPDase2. ADK activity increased the effects of exogenous ATP and ADP on IL-8 release in cells stimulated with a low concentration of poly (I:C). Indeed, the inhibition of ADK by Ap_5_A decreased IL-8 release promoted by these nucleotides. The Ap_5_A-dependent inhibition of IL-8 production triggered by exogenous ATP also suggests an involvement of NTPDase2, which provides a substrate for ADK through the hydrolysis of ATP to ADP. In contrast to NTPDase2, one might expect that such a significant potentiation of IL-8 release would not be possible if ADK was coupled with either NTPDase1, -3, or -8 which hydrolyze ADP and would therefore compete with ADK for this substrate. In agreement with the role of ADK in HT-29 cells, this enzyme present in lung epithelial cells also transiently maintains the increased concentration of ATP that can favor the activation of P2 receptors specific for that nucleotide [60]. ADK also promotes lymphocyte transendothelial migration by maintaining a significant level of ATP near the surface of these cells (halo ATP) [61].

We also observed that the ADK and NTPDase2 pair expressed in HT-29 cells makes the production of adenosine possible. Indeed, while NTPDase2 is required for ADP production from ATP, ADK converts two molecules of ADP to ATP and AMP. The latter is the substrate of ecto-5′-nucleotidase that converts it to adenosine which was detected in the cell supernatant of HT-29 cells (Figures 2(a), 2(c), and 2(d)). Adenosine production is of great physiological importance due to the large variety of functions played by the activation of P1 receptors in all tissues and cells, which includes key functions in the regulation of inflammation [62, 63].

Surprisingly, the presence of adenosine made possible by ADK and ecto-5′-nucleotidase did not affect IL-8 release in our conditions. We first anticipated that adenosine would affect IL-8 production in HT-29 cells. Indeed, in conditions of inflammation induced by TNF-*α*, the activation of the adenosine receptor A_3_ was previously shown to inhibit IL-8 expression through the inhibition of NF-*κ*B signaling pathways [37]. It is noteworthy that the secretion of IL-8 by adenosine appears to depend on experimental conditions in HT-29 cells as the adenosine receptor agonist NECA was also reported to induce a small IL-8 release under severe hypoxia via A_2B_ [38]. Nevertheless, while we found that NTPDase2 and ADK ectoenzymes could affect IL-8 release, our data also suggest that in other conditions, IL-8 secretion could also be potentially affected by the presence of ecto-5′-nucleotidase. In addition, as IL-8 is also produced by other epithelial cells in an ATP dependent fashion [64], such a regulation of nucleotide signaling by ectoenzymes is also possible in other cells.

Importantly, the presence of adenosine made possible by the presence of ADK and ecto-5′-nucleotidase could affect other functions in HT-29 cells. For example, adenosine increases HT-29 cells proliferation [39] via the activation of A_3_ receptor [36, 42]. The A_3_ receptor was the most expressed adenosine receptor in human colon cancer tissues and in colon cell lines such as HT-29 cells [42]. Interestingly caffeine, a P1 receptor antagonist, has been associated with a reduced risk of colorectal cancer in a number of case-control studies [38] which suggest an important role of adenosine and of ecto-5′-nucleotidase in colorectal cancer development.

## 5. Conclusions

HT-29 cells regulate adenine nucleotide levels by the combined action of NTPDase2, ADK, and ecto-5′-nucleotidase. This combination of ectoenzymes allows the generation of adenosine from secreted ATP while keeping ATP level high for a longer period of time. This also permits a sustained P2 receptor activation leading to IL-8 secretion which would normally be stopped rapidly by NTPDase2 if ADK was absent. These mechanisms of regulation of IL-8 release observed in this human cancer intestinal epithelial cell line might well play an important role in tumor progression as well as in the pathology of IBD and other related inflammatory disorders. If also present in primary cells, the underlining mechanism of IL-8 production identified in this work presents a new pathway that may be targeted in some of these associated diseases.

## Figures and Tables

**Table 1:** RT-PCR primers.

Gene	Forward primer	Reverse primer	Amplicon (bp)
*P2RY1 *	AAAACTAGCCCCCTGCAACT	GATCTGATGCCGGATGAACT	153
*P2RY2 *	CCACCTGCCTTCTCACTAGC	TGGGAAATCTCAAGGACTGG	163
*P2RY4 *	GCAGGGATATCATGGGTGAC	CCCAGGAAGGAACAGAAACA	109
*P2RY6 *	AGCTGGGCATGGAGTTAAGA	GCTGACTGGGACCTCTCAAG	139
*P2RY11 *	CCTCTACGCCAGCTCCTATG	CCTCTACGCCAGCTCCTATG	211
*P2RY12 *	TTTGCCCGAATTCCTTACAC	ATTGGGGCACTTCAGCATAC	192
*P2RY13 *	CCCCTGGTACACTTGGAAGA	TACAGAGGAGGGGGTGATTG	125
*P2RY14 *	TCTTTGGGCTCATCAGCTTT	TCCGTCCCAGTTCACTTTTC	213
*ENTPD1 *	GCCAGCAGAAAAGGAGAATG	TGGGACCTTGGAATCACTTC	159
*ENTPD2 *	TCAATCCAGCTCCTTGAACC	TCCCCAGTACAGACCCAGAC	167
*ENTPD3 *	TTGACCTCAGGGCTCAGTTT	TGAGGGGGTTCACTGCTTAC	159
*ENTPD8 *	ACTGGGCTACATGCTGAACC	GCACCATGAACACCACTTTG	107
*GAPDH *	CGACCACTTTGTCAAGCTCA	AGGGGTCTACATGGCAACTG	228
*AK1* *β* ^ a^	GGAATTCGACCATGGGCTGCTGCTC	GGAATTCGCAGCAGTGTGGGCTGTC	412
*AK1* *β*	ACAGGAGACACGGCAGGACGGGAC	CTCTTCTCCTTGCTGCACCTCC	385

^a^Taken from reference [46].

**Table 2:** Adenine nucleotide hydrolysis at the surface of HT-29 cells and protein extracts.

Activities	Intact cells (*n* = 4) [nmoles P_i_ *·*min^−1^ *·*well^−1^]	Cell lysates (*n* = 3) [nmoles P_i_ *·*min^−1^ *·*mg prot^−1^]
ATPase	7.5 ± 0.3	115 ± 4
ADPase	1.5 ± 0.01	38 ± 1
AMPase	6.5 ± 0.3	67 ± 3

The hydrolysis of ATP, ADP, and AMP as substrate was performed and the liberated P_i_ was determined by the malachite green assay as detailed in Section 2.
